# A Novel Task for the Investigation of Action Acquisition

**DOI:** 10.1371/journal.pone.0037749

**Published:** 2012-06-04

**Authors:** Tom Stafford, Martin Thirkettle, Tom Walton, Nicolas Vautrelle, Len Hetherington, Michael Port, Kevin Gurney, Pete Redgrave

**Affiliations:** Department of Psychology, University of Sheffield, Sheffield, United Kingdom; Katholieke Universiteit Leuven, Belgium

## Abstract

We present a behavioural task designed for the investigation of how novel instrumental actions are discovered and learnt. The task consists of free movement with a manipulandum, during which the full range of possible movements can be explored by the participant and recorded. A subset of these movements, the ‘target’, is set to trigger a reinforcing signal. The task is to discover what movements of the manipulandum evoke the reinforcement signal. Targets can be defined in spatial, temporal, or kinematic terms, can be a combination of these aspects, or can represent the concatenation of actions into a larger gesture. The task allows the study of how the specific elements of behaviour which cause the reinforcing signal are identified, refined and stored by the participant. The task provides a paradigm where the exploratory motive drives learning and as such we view it as in the tradition of Thorndike [1]. Most importantly it allows for repeated measures, since when a novel action is acquired the criterion for triggering reinforcement can be changed requiring a new action to be discovered. Here, we present data using both humans and rats as subjects, showing that our task is easily scalable in difficulty, adaptable across species, and produces a rich set of behavioural measures offering new and valuable insight into the action learning process.

## Introduction

### Theoretical Background

The ability of an agent to add new behaviours to its repertoire is a critical feature of intelligence, and crucial to the evolutionary success of species such as *homo sapiens*. A fundamental computational problem is for an agent to distinguish those things in the world it causes from those it doesn’t and, in so doing, discover what it is doing that is causing any particular outcome. We are interested in the mechanisms which allow an animal to identify that something it did caused an unexpected outcome, and thus to repeat and refine recent behaviour so as to home in on the causal elements of that behaviour. In other words, we are interested in how the brain extracts a fragment of the total space of all possible movements and stores it, making it available for subsequent selection as ‘something the animal does’ - an action with a known outcome.

This particular problem is understudied by the behavioural sciences. The most celebrated approach to action learning, operant conditioning, has often been focussed on rate of response as the critical dependent variable, and on variables that influence rate of response, *not* on how responses become identified and refined in the first place. The acquisition of actions is separate black from moderation of response frequency [2]. Consideration of the computational framework for understanding operant conditioning, reinforcement learning, makes this point clear [3], [4]. Although reinforcement learning focusses on the optimal algorithm for updating the value of different actions according to sampling of their consequences, it requires that all possible actions be defined in advance (i.e. that the representation of the ‘action space’ is known). In a review of the literature on operant conditioning Staddon & Niv [5] note that it is a ‘historical curiosity that almost all operant-conditioning research has been focused on the strengthening effect of reinforcement and almost none on the question of origins, where the behavior comes from in the first place.’

Our focus is more in line with that of Thorndike [1], and his famous experiments looking at cats learning to escape from a box. Thorndike recorded only escape time, but through this variable, showed how initial exploration by the animal was refined over repeated attempts until the key components, and only those, could be rapidly selected by the animal to affect a predicted change on the world, namely making possible the goal of escape. Thorndike’s paradigm captured the outcome of the process of searching motor space and refining exploratory movements into learnt actions.

We look in more detail at the relationship between exploratory movements and what is learnt, and so hope with our task to shine more light on this process of acquiring novel movement-outcome knowledge. This knowledge of a predictable outcome from a particular movement is key to our definition of an ‘action’. Motivations for exploration –– and sources of ‘behavioural variance’ which allow action discovery –– are of renewed interest [6].

The importance of exploration for learning has long been recognised in studies of human development [7], [8]. Bruner [9], [10] emphasised the intentional nature of action as critical to how skilled actions were learnt. In other words, even very early exploratory action is controlled by some anticipation of outcome. Piek [11] concludes that variability was essential for normal developmental motor learning, and that too little variability, as well as too much, could be associated with impaired learning.

We discuss below how our task allows the history of behavioural variance to be related to the acquisition of novel actions, and we present analyses that show a functional relationship between the amount of non-instrumental movement (‘exploration’) and subsequent competence (‘exploitation’).

### Criteria for Assessment of a New Task

Since in this paper we are not primarily introducing a new experimental result, but a novel experimental paradigm and a set of results associated with it, we introduce here a brief treatment of what qualities a novel paradigm should possess.

A novel behavioural paradigm should capture for our scientific inspection some element of behaviour, making it amenable to psychological and neuroscientific analysis. Although we want a task to capture some aspect of behaviour which consistently and significantly manifests in behaviour outside the lab –– to ‘carve nature at the joints’, as it were –– we also want the new paradigm to be simple enough to reflect the operation of a single aspect, or a related family of behaviours. The paradigm should give repeatable results which, while it is possible to relate these to existing theory, are also to some extent novel, in the sense that they confirm, contradict, or extend results from existing paradigms. Practically, the task would ideally be cheap and quick to run, and yield valid results even for non-naive subjects, enabling repeated measures designs.

### Outline of Task

The essence of the task is that the subject’s free movements are recorded, either via a manipulandum such as a joystick, or directly such as by using a touchscreen. Certain movements, henceforth ‘targets’, result in a sign or signal, henceforth the ‘reinforcement signal’. The task is to discover what characteristics of movement which evoke the reinforcement signal. The target may be defined in terms of absolute spatial position, in which case it is a ‘hotspot’, or in terms of a relative motion anywhere in absolute space, such as a line or circle. The target can even be related to the timing of the movement, e.g. onset or speed, regardless of its spatial characteristics. The success of many real-life actions will depend on all of these components. For different experiments with the task the reinforcement criteria can be defined in terms of one or more of these dimensions, so it is possible to investigate the discovery of different components of an action. When one target has been learnt the reinforcement criteria are simply changed and a new action has to be discovered. This therefore affords the requirements of repeated measures. Although participants are not naive to the whole task, they must learn a new action each time the target is changed.

Experiments reported in this paper investigate spatially defined targets. This gives the task a superficial similarity to the Morris water maze [12], with the proviso that it is possible to use the task with larger subjects (e.g. human and non-human primates), and that the timescale of the learnt movement is different from that of the water maze, as is the spatial scale of the movements learnt. A manipulandum is used for all experiments reported here.

**Figure 1:**
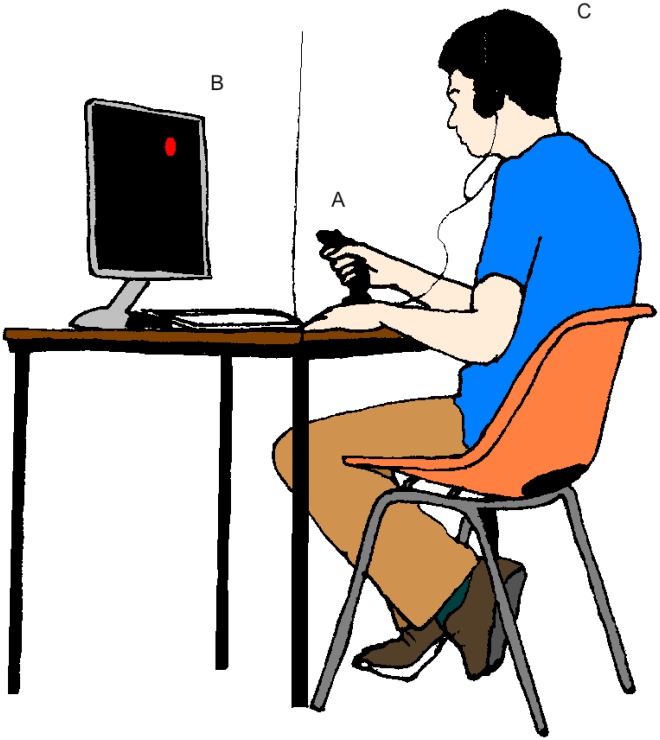
Experimental set-up for humans, showing (A) manipulandum, (B) location of visual signal of reinforcement, (C) participant engaged in task (not shown for rat subject).

**Figure 2:**
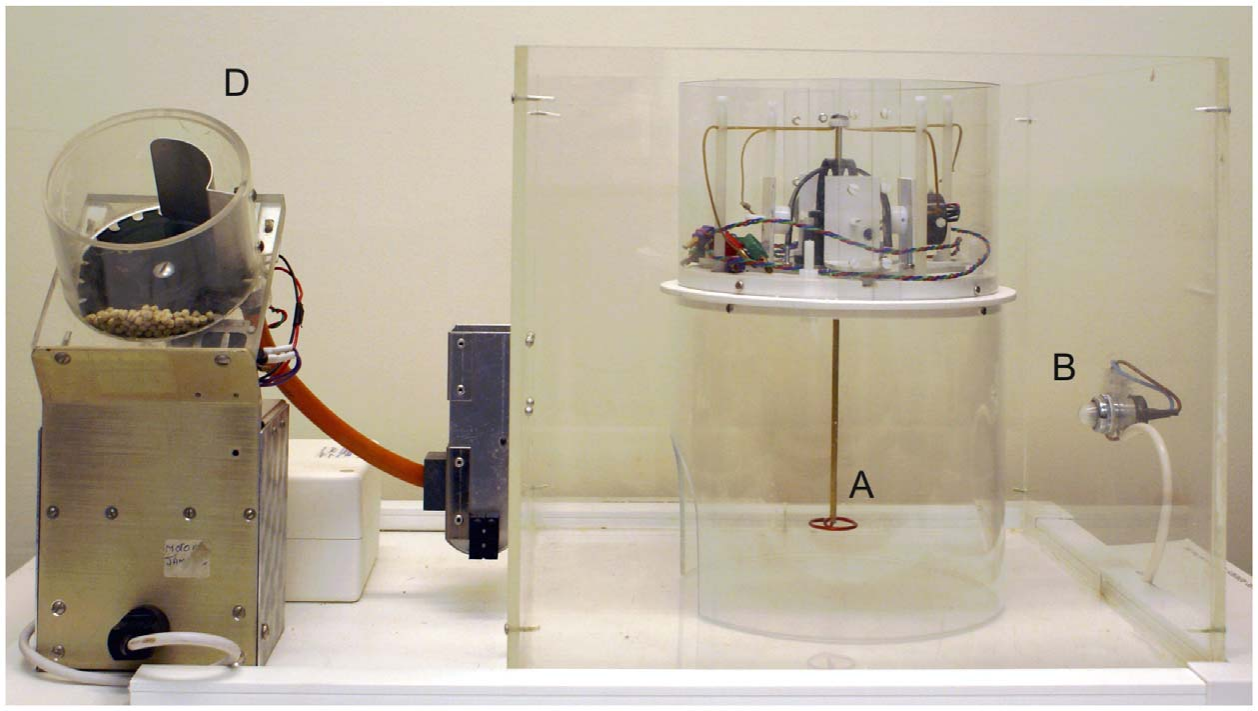
Experimental set-up for rats, showing (A) manipulandum, (B) location of visual signal of reinforcement, and (D) food hooper for delivery of rewards to maintain behaviour (not present for human subject).

Figures 1 and 2 show the apparatus for running the experiment with both human and rat participants respectively. Note that in the human set up the computer display is used only to deliver signals that the target motion has been made; it provides no visual feedback on the position of the joystick. For the rat version, a long-handled manipulandum hangs from the ceiling of the rat’s enclosure, to give it sufficient mechanical advantage. It can be moved with precision by the animal using a mouth or forepaw grip, or less precisely using a full body or tail swipe. Once moved, the rat joystick is engineered so that it maintains position rather than returning to the centre point. While a typical computer-literate human participant can be simply instructed to make exploratory motions with the joystick, rat participants require more direction. For the rat versions of the task we preconditioned the animal to associate the light with the subsequent delivery of the reward (over 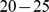 sessions) and then shaped the animal’s behaviour by initially reinforcing any movement of the joystick (for 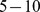 sessions) and only then assessing subsequent attempts to acquire a more selective target. This pre-training takes the place of instruction in the human, allowing subjects of both species to begin the task with an understanding of the general task, but not the specific target. A direct comparison of the learning process for human and rat subjects cannot be freely assumed. It remains an open empirical question whether it is possible to use the task in a similar way to investigate common processes underlying action acquisition.

## Results

### Characterising Behaviour

**Figure 3:**
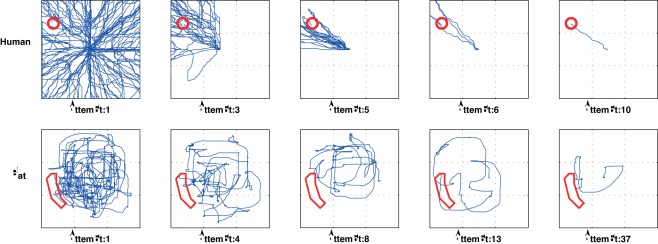
Movement traces (blue) for a spatial target (outlined in red) for typical (a) human and (b) rat participants.



Figure 3 shows typical continuous traces from both human and rat subjects as they initially explore, and then refine, their movements so as to ‘home in’ on a spatially defined target. Note the similarity in the plots. Although rats take longer to refine movements into a stereotyped action, the similarity in the progression of behaviour in this spatial version of the task suggests that we are tapping into a similar process in both species that relies on similar underlying machinery of action-discovery. Qualitative support for this suggestion is given in the subsequent analyses presented below.

### Validity and Reliability of Measures

#### Learning rate analysis

Within each session, for both human and rat participants, performance improves –– a key dynamic of any putative learning phenomenon. Analysis of average performance shows that learning rates can be approximated by the power law of learning [13], [14], having the form

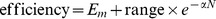
(1)Where *efficiency* is some measure of performance (with lower values representing better performance), 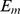 is a minimum, *range* the difference between the initial and asymptotic value of the performance measure, 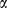 is the parameter which defines speed of learning, and 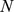 is the number of trials.

**Figure 4:**
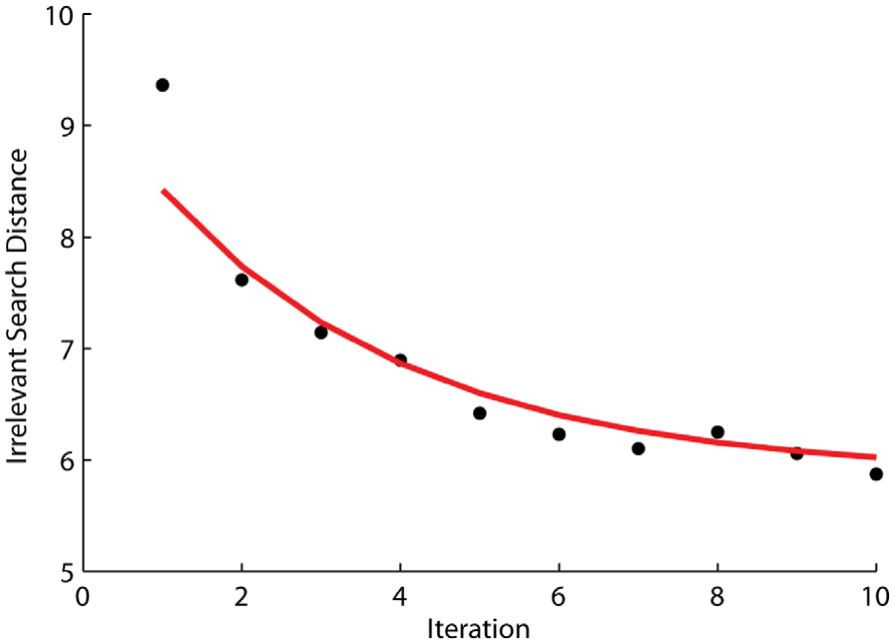
Human performance follows the power law of learning. N = 30.

Performance of human subjects improves with practice. Figure 4 shows average performance data over 10 trials (N = 30). This is fitted closely by a power law (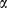 = 0.31, SSE 1.03).

**Figure 5:**
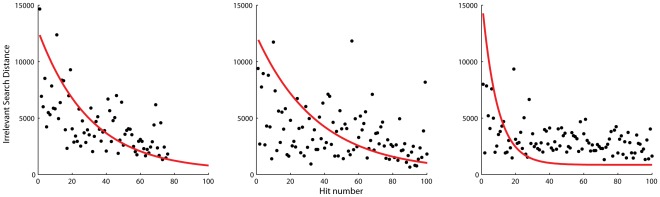
Rat performance follows the power law of learning. N = 6.

Rat performance is more variable. By comparing early and late training sessions (shown in Figure 5, 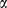 values of 0.03, 0.03 and 0.11) we can see that some task learning does occur, but that within-session learning is the major determinant of performance –– each time the animal attempts the task significant learning is occurring. By changing the target we ‘reset’ the task so that the performance measure is a relatively pure index of within-task learning.

#### Difficulty

**Figure 6:**
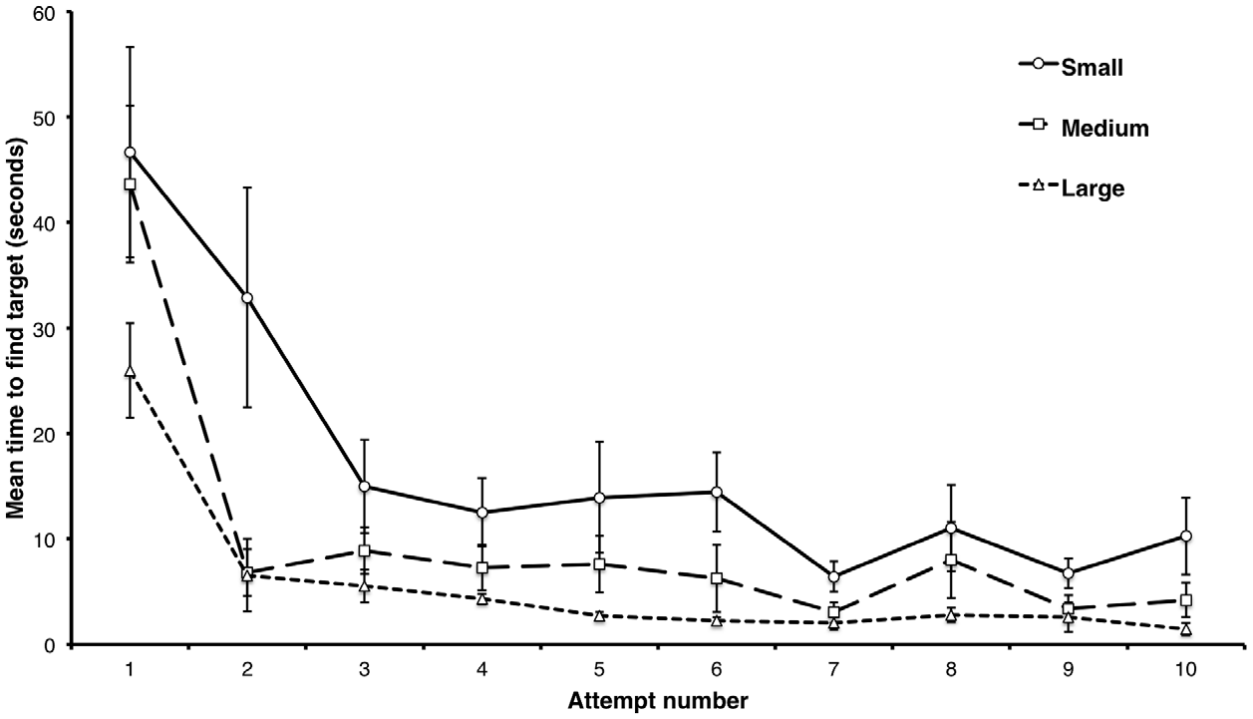
Task difficulty can be calibrated by adjusting target size Performance shown for different targets. Human subjects (N = 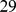).

Figure 6 shows that the task is easily scalable in difficulty, in this case by adjusting the size of a spatially defined target. This means the task has the potential to be individually calibrated for difficulty, so that all subjects can be recorded while attempting the task at the limit of their abilities. Thus the task can be adapted to different populations, for example children or groups with neuropsychological conditions.

### A Lens on Action Discovery

#### Exploration and exploitation

**Figure 7:**
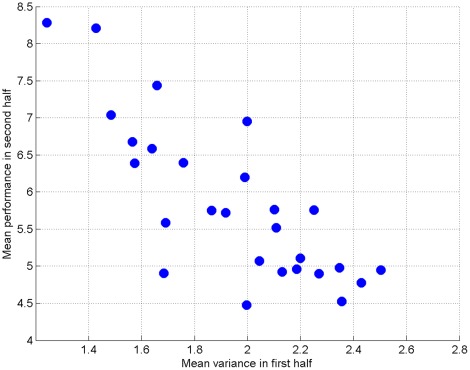
Greater exploration associated with improved performance, across different participants.

**Figure 8:**
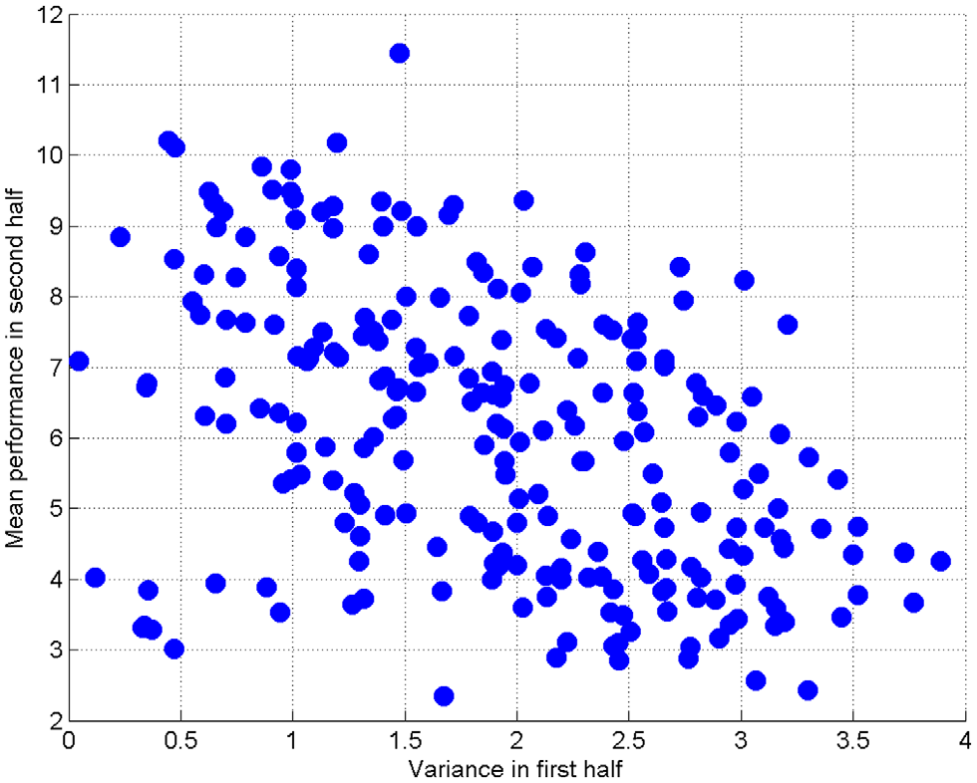
Greater exploration associated with improved performance, across different sets of trials.

A prediction from learning theory is that greater exploration is associated with improved final performance [6]. We assessed this by calculating the *variability* in performance for the first half of trials, and comparing it with the *average* performance in the second half of trials. Path length from the beginning of a trial until the target was reached was used as a proxy for performance. This was positively skewed so all distances were log transformed. The average path length for the first 5 and last 5 of 10 trials was used as a measure of first half and second half performance respectively. The standard deviation over the first 5 and last 5 of 10 trials was used as a measure of variability. Looking at the average performance and variability for each individual subject, those that were more inconsistent at the beginning of learning were better in the second half (see Figure 7). This effect also holds *within* subjects, so that for individual targets which were learnt over ten trials, those for which subjects explored more initially also showed better performance subsequently (Figure 8). The average correlation between first half performance and second half performance, across 30 human subjects, was 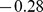 (one sample t-test, different from zero with 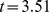, 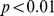).

The same pattern holds for rats. Across different individuals, those who explore more in the first half of each training session perform better in the second half. The average correlation between first half performance and second half performance was negative (n
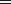
6, mean correlation = 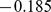; one sample t-test, different from zero with 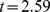, 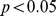). Comparing across sessions, the pattern also holds: when an animal explores more in the first half it tends to perform better in the second half (correlation = 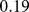, 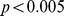).

## Discussion

### Benefits

The task provides a rich set of behavioural measures. The moment-by-moment recording of the discovery of actions can give insight into the micro-features of action learning. For example, one issue we have considered is the extent to which accidental or coincidental movement features that are present during a first successful movement will be preserved and reinforced. We have supposed that unexpected events provoke inspection of a limited segment of the record of motor output, the equivalent to the eligibility trace in reinforcement learning [15]. Identification of the time window, relative to an outcome, for which non-causal movement features are not eliminated from an action as it is refined may be revealing of the temporal extent of this record of motor output. The manipulation of delay between target-movements and reinforcement signal may also be revealing of these internal processes.

The rich set of behavioural measures can also be converted into robust statistics which show the progression of learning throughout a batch of trials. Candidate statistics include total length of movement in between reinforcement signals, time taken to complete movement and various measures of movement complexity and efficiency.

A prime benefit of the task is that it does not take long to perform and once a particular target has been learnt the target can be switched so that the same non-human animal or human participant can repeat the process of action learning. This allows experiments with repeated-measures designs (which allow analyses of greater statistical power) while reducing greatly the expense and time-cost of experimentation in comparison to those tasks that require fresh subjects for each batch of trials.

The task enjoys a number of other practical benefits. It is scalable in difficulty, simply by altering the required precision of the target. For example, in the spatial version of the task this corresponds to the size of the hotspot. This means that task performance can be equated across different populations (e.g. patients versus controls, lesioned and non-lesion animals).

### A Distinct Kind of Learning

#### Distinct from operant conditioning and reinforcement learning

Our task has a different focus from those that look at the attachment of value to actions. The way outcome value (and aspects of outcome delivery) determines the distribution of effort once eliciting actions have been discovered is the focus of operant conditioning experiments and reinforcement learning theory. We, instead, focus on the process of action discovery. This is a problem which necessarily must be solved if the problem of how to value actions is to be solved. The problem is that of identifying what movements of the entire space of possible movements create distinct outcomes in the world and so are worth storing and repeating as actions. Reinforcement learning [3] gives a principled computational account of the credit assignment problem in operant conditioning, but assumes a given set of actions to which credit can be optimally assigned. Our task aims to address this additional requirement of action-learning, that of identifying what movements are actions.

It is worth noting that the primary technology of operant conditioning research, the Skinner box, makes automatic the recording of response rate at the cost of making invisible the processes leading up to the selection of the response. Thorndike’s procedure required a ‘stamping out’ of all behaviours which did not allow the cat out of the box, and is close to the aspects of action learning upon which we want to focus. Skinner’s procedure involves familiarising the animal with the box, so that other behaviours have already diminished and thus the ‘attentional problem’ is solved for the animal. Only the lever, the novel element, is the subject of exploration and so this exploratory element is minimised and controlled for, to allow the experimental focus on response rates alone (this is discussed by Skinner himself [16]). Since then rate of response has been a primary focus of animal learning research although note that subsequent behaviourist research has used other variables such as inter-reinforcement interval or inter-response time). The use of rate as a metric suggests an interest on the part of the experimenter in those behavioural events that occur after a response has been acquired. Indeed laboratory practices are often geared towards reducing the period of response acquisition as much as possible. The technique of shaping [17], [18] and the use of nose poke responses in place of more traditional operandum-focused responses [19] are both motivated by a desire to speed up the process of acquisition and allow researchers to concentrate on recording the rate of elicitation of the fully formed response. However, in spite of the implied focus on post acquisition behaviour, rate is used as a metric in the study of response acquisition [17], [20], [21]. There are obvious practical benefits to this because it allows researchers to employ widely documented, universally understood experimental techniques; however, the use of rate as a metric brings with it an unavoidable limitation: rate is only indirectly related to the efficiency with which an action is performed because it is also a measure of choice and not just of performance. It is, therefore, difficult to differentiate between a fully formed response and one that is midway through acquisition. Researchers are often forced to identify acquisition as the point at which rate exceeds an arbitrarily determined threshold. True performance metrics –– even ones as simple as the escape time metric employed by Thorndike [1] –– give a much better representation of the efficiency of an action and they do not necessitate the use of thresholds because they directly describe a particular parameter of the current state of acquisition. Skinner showed that the relation between effects and actions could be systematically studied, but Thorndike’s demonstration that from many movements the critical components for causing a particular outcome could be identified and refined into a stereotyped action has been relatively ignored [5].

Note that the reinforcement signal in this task is not a primary reward. Although overall behaviour may be rewarded, either by extrinsic rewards such as food or drink, or by intrinsic rewards such as novelty and satisfaction of curiosity, or by associated secondary reinforcers of both of these, it is the relationship between action and reinforcement that is tightly locked in time, whereas rewards (i.e. at the end of the experiment, or after a certain amount of reinforcement has been collected) are less tightly bound to reinforcements (and even less so to actions).

#### Distinct from motor learning

There is a considerable literature which deals with the topic of motor learning and the computational theory of optimal control, in the engineering sense [22]. It is worth noting that the problems upon which motor control theories tend to be based involve a single action, or small set of actions, which are ‘declared’ by the experimenter. Braun and Wolpert distinguish the ‘parametric’ learning studied in most of these tasks from the learning which covers wider aspects of the purpose of the task, which they terms ‘structural’ learning [23]. By providing continuous feedback on motor performance the motor learning studied in these tasks may be understood computationally as a form of supervised learning [22]. The Thorndikian process of action discovery is thus avoided. The tasks used for such studies of motor learning, in our view, focus on the ‘how’ of motor control, rather than the ‘what’ which is the subject of our interest. In biological terms this relates to the parameterisation of an action so that it may be efficiently and correctly performed (i.e. timing and force of muscle contractions). Studies of motor learning tasks have found a heavy involvement of the cerebellum in this process [24], [25]. An aspect which is not accounted for by cerebellar-orientated theories of motor control, and which is covered by Braun and Wolpert’s ‘structural learning’, is the novel action acquisition we hope to capture with our task.

#### Distinct from action-outcome learning

Tony Dickinson has provided a compelling and thorough account of what he has called ‘Action-Outcome’ learning [26], [27]. This action-outcome learning is contrasted with habit learning, and it is part of a goal-directed learning system in which the outcome associated with an action is integral to its representation. We would view action learning of the sort studied in our task as necessary but not sufficient for this kind of action-outcome learning. In other words, Dickinson and colleagues have shown convincingly that rats can select actions according to the outcome associated with them, an important cognitive capacity which is beyond the reach of mere operant conditioning of actions (the ‘habit system’). Both these systems, we claim, are predicated upon the discovery of novel actions. Once discovered, actions can both be reinforced by their consequences, or associated with outcomes.

One test of the distinctiveness of action-outcome learning in the Dickinsonian sense from action-discovery as present in our task may be the sensitivity of performance to delays in the reinforcement signals. Free-operant acquisition has been shown to be robust to delays of up to 32 seconds in the rat [28], although shorter delays of around 2 seconds can have dramatic effects on the performance of instrumental actions in both rats and humans [29], [30]. To our knowledge there are no direct tests of the effect of delay on action-outcome learning (we thank an anonymous reviewer for pointing this out), but it is reasonable to suspect that it would have a timing sensitivity comparable to that of free-operant acquisition. This relative insensitivity, compared to the timing sensitivity of action-discovery in our new task, may provide a signature which we can use to compare the two.

#### A requirement of action learning

As discussed, we view intrinsically motivated action learning as a necessary, but not always accounted for, component for the above kinds of learning to occur. In Staddon and Niv’s [5] terms we are focussing on the ‘origins and refinement of behavioural variety’. We see this as in the tradition of Thorndike [1], in that the emphasis is on exploration as a route to action discovery. Variation between movements is required to identify which components of previous behaviour were truly causal in provoking an outcome, and which were merely coincidentally associated. In Thorndike’s task the question of value (“how much was that action worth?”) is deprioritised (escaping the box is unambiguously very high value). Rather the question of the moment is “what was it I just did?”. As discussed, reinforcement learning does have an account of how credit is assigned to previous actions, but this framework assumes that the relevant actions are given. Our concern is how the brain identifies these relevant actions. Recent research has shown that response variability, as well as frequency, can be directly reinforced [31], [32], and, further, that variability systematically changes with changes in reinforcement [33]. This suggests that an underexplored component of operant conditioning may be the variability of responding and the way such variability functionally supports action acquisition.

### A Window on Intrinsically Motivated Learning

Our task provides a window on how exploration may be related to action learning. Although the arena of action learning is narrow and directed relative to the very broad space of all that might be considered ‘intrinsically motivated learning’ [6], we feel it still has some important lessons to impart. It is difficult to argue that any behaviour is entirely intrinsically motivated, where this is defined as being entirely separate from exogenous rewards, but it may still be possible to investigate aspects of behaviour which do not immediately and directly provoke exogenous rewards. An example of such an aspect is the exploration in our action acquisition task. The exploration-performance relation shown above is an example of how the task can be related to the core issues of the idea of intrinsic motivation.

Specifically, the task allows us to ask questions of the nature of the representations formed during intrinsically motivated action discovery. The paths formed by the animal in the course of learning an action are a rich data set, which should allow us to ask what elements of behaviour are reinforced –– are the speed, final position and/or trajectory of successful movements retained? In addition, through analyses and the manipulation of factors such as reinforcement signal, reinforcement timing and exploration strategy we hope to be able to uncover a richness of information about the representations formed during action learning that has not hitherto been available.

## Materials and Methods

### Ethics Statement

All human work was approved by the University of Sheffield, Department of Psychology Ethics Sub-Committee (DESC). All this was carried out in accordance with the University and British Psychological Society (BPS) ethics guidelines. Written informed consent was obtained from all participants involved.

Care of animals: all animal husbandry and experimental procedures were performed in the UK with Government Home Office approval under section 5(4) of the Animals (Scientific Procedures) Act 1986. Experimental protocols also received prior approval according to University of Sheffield ethics guidelines.

### The Task with Human Participants

The experiments were run using Matlab (Version 2007) with the Psychophysics Toolbox extension [34]–[36]. A commercial joystick (Logitech extreme 3D pro joystick, P/N: 863225-1000) was used as the manipulandum, with inputs sampled at 1000 Hz. Code for the experimental programmes is available upon request. The search space was defined as a square that was 1024 by 1024 units in size, which corresponded to the limits of the joystick’s travel (the joystick movements were physically restricted by a square aperture at the base of the stick). Movements of the joystick mapped on to movements within the search space in a 1 to 1 fashion, with the joystick starting in the centre of the search space at the beginning of each trial. Once released from the grip of a participant, the joystick was able to return to the centre of the search space within a tolerance of 10 units, by virtue of a built-in spring mechanism.

Different sizes of reinforced area (‘hotspots,’ which for this task are circles defined in the search space) were tested during development and piloting of the task. The size was eventually set to occupy 0.91% of the overall search space based on finding a balance between making the task sufficiently difficult to provide useful data and the practical limitations of running multiple trials that were not time-limited. At the beginning of every new trial, the centre of the hotspot was positioned randomly on an annulus placed centrally within the search space. The inner edge of the annulus was exactly 1 times the diameter of the hotspot from the centre of the search space. The outer edge of the annulus was exactly 1 times the radius of the hotspot from the edge of the search space. The reason for these dimensions was to ensure that the hotspot never overlapped the central starting point or the outer edge of the search space. Any movement of the joystick into the hotspot region of the search space was defined as a hit and resulted in a whole screen flash of 17 ms.

In the ‘continuous’ version of the task (see below), generating a single hit was not sufficient to bring an end to a trial. Instead, a criterion was used to determine whether a participant had located the hotspot (a.k.a an ‘escape criterion’, in reference to Thorndike’s cats). The escape criterion was defined as the number of hits required within 1 second in order to bring an end to a trial. Like hotspot size, the escape criterion was set using information gained from pilot tests in order to balance task difficulty (more hits per second meant the threshold was harder to meet) against better verification of learning (more hits per second requires a participant to demonstrate better learning of the hotspot location). The criterion was set at 15 hits per second. From an individual participant’s perspective the aim in a given trial was, therefore, to find the hotspot and try to maintain the position of the joystick over this region until having achieved 15 hits in a second. Participants sat at a desk in front of the joystick and a 19 inch computer monitor. Before starting the experimental program, the task was briefly described verbally with the task goal being phrased in terms of “finding the correct position to place the joystick in” rather than, say, “search for the correct location”.

### The Task with Rat Subjects

Rats completed a similar version of the task, using a specially constructed ‘rat joystick’, which hung from the ceiling of the animal’s enclosure (see Figure 2). There were two major difference from the human version of the task. Firstly, movement of the joystick into the target area/hotspot turned on the box light (Figure 2B). After the light had been on for 1 cumulative second a food reward would be delivered with a five second delay. The food reward is necessary to maintain the animal’s behaviour; the five second delay is so that task performance is most immediately guided by the light, rather than by the primary reinforcer of the food. Whilst the rat is feeding the joystick position is moved to a new random position. The second major difference from human participants is that the rats underwent a pre-testing training regime of a) sensory preconditioning, where the light was associated with food delivery via classical conditioning, and b) shaping, where the animal was taught to associate progressively more precise movements of the joystick with the light reinforcer. Typically a rat would spend 30 mins each day in the experiment, with the target staying the same for the entire session and changing to a new random position each day. Code for running the experiments is available upon request.

### Metrics of Performance

We experimented with a number of metrics of task performance.The two main ones we use here are total time to locate target (‘search time’) and the total irrelevant distance travelled, defined as the path length of manipulandum travel on a trial which is in excess of the length of the direct line between starting position and target position. For most experiments these two metrics are tightly correlated, only diverging when movement speed changes without the trajectory changing or comparable cases. Note that the irrelevant distance metric is insensitive to changes in speed, and is most relevant to versions of the task, as reported here, where the target is defined in simple spatial terms. Because of these limitations we have not focussed exclusively on it, but also reported results using the search time metric.

### Continuous vs Iterated Version of the Task

Consider two parameters of the task: the number of attempts that the participant gets with a particular target before a new target is selected, and the escape criterion (the action(s) defined as the criterion of having found the target). If multiple attempts are allowed we have an ‘iterated’ version of the task, in which it is possible to observe the acquisition and refinement of the correct movement over multiple attempts (with or without different starting positions). If the escape criterion is more strict than a single hit (e.g. a single entry into the target area) then it is possible for the participant to refine their knowledge of the target without returning to a starting position and needing to evoke a whole movement (i.e. they can reverse their current movement and repeat their most recent actions). A version of the task with a stringent escape criterion and only one attempt for each target would be a ‘continuous’ version of the task, rather than ‘iterated’. We report results from both versions of the task here (iterated versions for the data shown in Figures 3, 4, 6, 7 and 8; continuous version for the data shown in Figure 5). We believe that the continuous version is more informative of the link between reinforcement signal and target representation, while the iterated version is more informative of nature of the action representation as it develops (Walton, Thirkettle, Gurney, Redgrave and Stafford, in preparation).

## References

[1] Thorndike E (1911). Animal intelligence..

[2] Redgrave P, Gurney K (2006). The short-latency dopamine signal: a role in discovering novel actions?. Nature Reviews Neuroscience.

[3] Sutton R, Barto A (1998). Reinforcement learning - An introduction..

[4] Woergoetter F, Porr B (2007). Reinforcement learning.. Scholarpedia.

[5] Staddon J, Niv Y (2008). Operant conditioning.. Scholarpedia.

[6] Baldassarre G, Mirolli M Intrinsically Motivated Learning in Natural and Artificial Systems..

[7] Gibson E (1988). Exploratory behavior in the development of perceiving, acting, and the acquiring of knowledge.. Annual Review of Psychology.

[8] Thelen E (1989). The (re) discovery of motor development: Learning new things from an old field.. Developmental Psychology.

[9] Bruner J (1973). Organization of early skilled action.. Child Development.

[10] Kalnins I, Bruner J (1973). The coordination of visual observation and instrumental behavior in early infancy.. Perception.

[11] Piek J (2002). The role of variability in early motor development.. Infant Behavior and Development.

[12] Morris R (1984). Developments of a water-maze procedure for studying spatial learning in the rat.. Journal of Neuroscience Methods.

[13] Newell A, Rosenbloom P (1981). Mechanisms of skill acquisition and the law of practice.. Anderson J, editor, Cognitive Skills and their Acquisition, Lawrence Erlbaum.

[14] Ritter F, Schooler L (2001). The learning curve.. Smelser N, Baltes P, editors, International Encyclopedia of the Social & Behavioral Sciences, New York: Elsevier.

[15] Singh S, Sutton R (1996). Reinforcement learning with replacing eligibility traces.. Machine Learning.

[16] Skinner B (1969). Contingencies of Reinforcement: A Theoretical Analysis..

[17] Lattal K, Gleeson S (1990). Response acquisition with delayed reinforcement.. Journal of Experimental Psychology: Animal Behavior Processes.

[18] Peterson G (2004). A day of great illumination: B.F. Skinner’s discovery of shaping.. Journal of the Experimental Analysis of Behavior.

[19] Schindler C, Thorndike E, Goldberg S (1993). Acquisition of a nose-poke response in rats as an operant.. Bulletin of the Psychonomic Society.

[20] Snycerski S, Laraway S, Huitema B, Poling A (2004). The effects of behavioral history on response acquisition with immediate and delayed reinforcement.. Journal of the Experimental Analysis of Behavior.

[21] Snycerski S, Laraway S, Poling A (2005). Response acquisition with immediate and delayed conditioned reinforcement.. Behavioural Processes.

[22] Wolpert D, Ghahramani Z, Flanagan J (2001). Perspectives and problems in motor learning.. Trends in Cognitive Sciences.

[23] Braun D, Aertsen A, Wolpert D, Mehring C (2009). Motor task variation induces structural learning.. Current Biology.

[24] Jueptner M, Weiller C (1998). A review of di®erences between basal ganglia and cerebellar control of movements as revealed by functional imaging studies.. Brain.

[25] Diedrichsen J, Verstynen T, Lehman S, Ivry R (2005). Cerebellar involvement in anticipating the consequences of self-produced actions during bimanual movements.. Journal of Neurophysiology.

[26] Adams C, Dickinson A (1981). Instrumental responding following reinforcer devaluation.. The Quarterly Journal of Experimental Psychology: Section B.

[27] Dickinson A, Balleine B (2000). Causal cognition and goal-directed action.. Heyes C, Huber L, editors, The evolution of cognition, The MIT Press.

[28] Dickinson A, Watt A, Gri±ths W (1992). Free-operant acquisition with delayed reinforcement.. The Quarterly Journal of Experimental Psychology: Section B.

[29] Dickinson A (2001). The 28th Bartlett Memorial Lecture. Causal learning: An associative analysis.. The Quarterly Journal of Experimental Psychology: Section B.

[30] Elsner B, Hommel B (2004). Contiguity and contingency in action-effect learning.. Psychological Research.

[31] Ross C, Neuringer A (2002). Reinforcement of variations and repetitions along three independent response dimensions.. Behavioural Processes.

[32] Paeye C, Madelain L (2011). Reinforcing saccadic amplitude variability.. Journal of the Experimental Analysis of Behavior.

[33] Neuringer A, Jensen G (2010). Operant variability and voluntary action.. Psychological Review.

[34] Brainard D (1997). The psychophysics toolbox.. Spatial Vision.

[35] Pelli D (1997). The videotoolbox software for visual psychophysics: Transforming numbers into movies.. Spatial Vision.

[36] Kleiner M, Brainard D, Pelli D, Ingling A, Murray R (2007). Whats new in psychtoolbox-3.. Perception.

